# A functional polymorphism T309G in MDM2 gene promoter, intensified by *Helicobacter pylori* lipopolysaccharide, is associated with both an increased susceptibility and poor prognosis of gastric carcinoma in Chinese patients

**DOI:** 10.1186/1471-2407-13-126

**Published:** 2013-03-18

**Authors:** Xiaolin Pan, Yuqin Li, Jin Feng, Xiaoyong Wang, Bo Hao, Ruihua Shi, Guoxin Zhang

**Affiliations:** 1 Department of Gastroenterology, the First Affiliated Hospital of Nanjing Medical University, Nanjing 210029, China; 2 Department of General Surgery, the First Affiliated Hospital of Nanjing Medical University, Nanjing, China; 3 Department of Gastroenterology, Changzhou No.2 People’s Hospital, Affiliated with Nanjing Medical University, Changzhou 213000, China

**Keywords:** Helicobacter pylori, Gastric carcinoma, MDM2, SNP, Lipopolysaccharide

## Abstract

**Background:**

Studies on the association between *MDM2* SNP309 (T > G) and gastric cancer have reported conflicting results. Thus, the aim of this study was to investigate whether *MDM2* SNP309 is associated with susceptibility and prognosis of gastric carcinoma in Chinese patients.

**Methods:**

Total of 574 gastric carcinoma cases and 574 age- and sex-matched healthy controls were included. *MDM2* polymorphism was detected by PCR- RFLP and infection of *Helicobacter pylori* (*H. pylori*) by a validated serology test. The functionality of *MDM2* SNP309, with or without *H. pylori* lipopolysaccharide (LPS), was examined by dual-luciferase assay. Kaplan-Meier survival curves were used to evaluate survival. Additional, a meta-analysis was conducted to verity the findings.

**Results:**

*MDM2* SNP309G/G genotype was associated with an increased risk of gastric carcinoma when compared with T/T genotype or T carriers (both *P* < 0.01), and a joint effect between *MDM2* SNP309G/G and *H. pylori* infection was observed to intensify gastric carcinoma risk. SNP309G/G was identified as an independent marker of poor overall survival of carcinoma. In vitro, the luciferase assay further showed an increased transcriptional activity of SNP309G allele compared with SNP309T allele, and the function of polymorphism T309G in *MDM2* gene promoter was intensified by *H. pylori* LPS. Pooled results from the meta-analysis confirmed that SNP309G/G genotype had a significantly increased risk of gastric carcinoma compared with T/T genotype or T carriers, consistent with the case–control findings.

**Conclusions:**

*MDM2* SNP309G allele is associated with an increased risk and poor prognosis of gastric carcinoma in Chinese patients. Additional, there is a joint effect of MDM2 SNP309G/G allele and *H. pylori* infection on gastric carcinoma development, which may attribute to *H. pylori* LPS.

## Background

The *p53* is well-known to be crucial in tumor prevention, which is activated by stress and oncogene activation as a transcription factor, and give rise to lots of cellular responses, such as cell cycle arrest, apoptosis, and senescence [1]. More than 50% of human carcinomas exist *p53* mutation or deletion [2]. *MDM2* is an oncogene, encoding E3 ubiquitin ligase, which negatively regulates p53 transcriptional activity and protein stability [3], as it has been reported that the embryonic lethality of *MDM2* null mice is caused by the uncontrolled activity of p53 and rescued by its deletion [4]. MDM2 overexpression could inhibit p53 function, and make damaged cells avoid the cell cycle checkpoint control and become carcinogenic [5,6]. In addition, there is a negative auto-regulatory feedback loop between MDM2 and p53, which plays an important role on regulating cell cycle progression, apoptosis and senescence [3].

A T-to-G single nucleotide polymorphism (SNP) is located 309 base pairs in promoter of *MDM2*, which known as *MDM2* SNP309 (T > G), has been found to enhance the binding of the transcriptional activator specificity protein 1 (Sp1) to the P2 promoter of *MDM2*, resulting in an increased *MDM2* transcription and MDM2 protein expression, and a weaken of the p53 tumor suppression function [7]. It’s important that the *MDM2* SNP309G allele has been shown with an increased risk for the development of some tumors which express wild-type p53 [8-11].

*H. pylori* is a gram-negative pathogen that colonizes approximately 50% of the world’s population, and is identified as a cause of gastric carcinoma development [12-14]. LPS of *H. pylori* is the constituent of its outer membrane, which has been reported to have the ability of promoting colonization of the mucus layer adjacent to the gastric epithelial surface [15], and enhancing proliferation and progression of gastric carcinoma [16,17].

Gastric carcinoma is the second most common reason for carcinoma-related deaths, accounting for more than 1 million deaths every year worldwide [18]. Several studies have investigated the association between *MDM2* polymorphism and gastric carcinoma susceptibility [19-23]. However, these studies have produced inconsistent and inconclusive results. In our previous study, we reported that the *MDM2* SNP309 (T > G) was associated with gastric carcinoma in Chinese patients, especially those with *H. pylori* infection [23]. However, that study was of small sample size and did not address any functional relation between *MDM2* SNP309 (T > G) and gastric carcinogenesis. Moreover, there has been no report on the association between *MDM2* SNP309 (T > G) and survival of gastric carcinoma patients in Chinese patients. Therefore, our aim is to investigate whether *MDM2* SNP309 (T > G) is associated with susceptibility and prognosis to gastric carcinoma in Chinese patients.

## Methods

### Study population

574 gastric carcinoma patients and 574 healthy controls were included. All subjects reside in Jiangsu Province of China, and are unrelated ethnic Han Chinese. Patients were consecutively recruited from July 2005 to July 2009, at the First Affiliated Hospital of Nanjing Medical University. All cases were those newly histologically diagnosed with gastric carcinoma without previous chemotherapy or radiotherapy, which was staged and classified according to the American Joint Committee on Cancer Staging Manual, and Lauren’s classification, when diagnosed [24,25]. All healthy controls were recruited from individuals living in the same residential areas who attended routine medical examination at the same hospital without no abnormal findings at the examination and were age- (±5 years) and sex-matched to the cases. All subjects who had a history of tumors including gastric or other tumors or had undergone eradication treatment for *H. pylori* were excluded.

Venous blood (2 ml) was collected from each subject at the entry of the study for the genotyping and detection of *H. pylori* infection. Gastric carcinoma patients were followed-up every 3 months by telephone or patient’s reexamination to the hospital to update the progress of the disease.

This study has been approved by the Ethics Committee of the First Affiliated Hospital of Nanjing Medical University, and the written informed consent was obtained from all subjects, and the institutional ethics committees approving this research comply with acceptable international standards (the Treaty of Helsinki).

### Detection of H. pylori infection

The indirect solid phase immunochromatographic assay was applied to detect the infection of *H. pylori* according to its IgG antibodies (Genelabs Diagnostics Pty Ltd, Singapore), which has been validated in Chinese population with an accuracy of 92.3% [26].

### Genotype analysis

Genomic DNA was extracted by proteinase K digestion and followed by phenol–chloroform extraction and ethanol precipitation from a leukocyte pellet. Polymerase chain reaction restriction fragment length polymorphism (PCR-RFLP) was used to determine the genotype of *MDM2* SNP309 [23]. In brief, the primer sequences were 5P-CGCGGGAGTTCAGGGTAAAG-3P (forward) and 5P-AGCTGGAGACAAGTCAGGACTTAAC-3P (reverse), which generated the 237-bp fragment. The PCR product was then digested by *MSPA1I* (New England Biolabs, Bevely, MA, USA). The wild-type (SNP309T) allele produces a single 237-bp fragment and the variant (SNP309G) allele produced two fragments of 189- and 48 bp. Genotyping was performed without knowledge of the case or control status. The gel images were read independently by two research assistants. If a consensus was not reached on the tested genotypes, then the genotyping was repeated independently until a consensus was reached. In addition, to validate the RFLP method, 100 (50 from cases and 50 from controls) PCR products were selected randomly for direct sequencing with ABI 3700 sequencer, and the concurrence rate of these two methods was 99%.

### Construction of reporter plasmids

Because the association between MDM2 SNP309 (T > G) and gastric carcinoma susceptibility, we then determined whether this polymorphism had an effect on gene expression in vitro. The MDM2 promoter-luciferase reporter plasmids containing either 551 T or 787 G sequence were prepared by amplifying the 237 -bp MDM2 promoter region by using primers with restriction sites. The primers were 5P-GACGCTAGCTTTGTCG-CGCAGTTTCCACCG-3P (forward) and 5P-CGCGAAGCTT CTTCTTG-CTCCATCTTTCC-3P (reverse), including the NheI and HindIII restriction sites. We confirm the amplified fragments by sequencing. The amplified fragments and pGL3-basic vector (Promega, Beijing, China) were cleaved by using the NheI and HindIII enzymes (TaKaRa Biotech Co., Dalian, China). After the fragments were cloned into the pGL3-basic vector, the vectors were sequenced to confirm that there were no wrong.

### Cell culture

Human gastric cancer cell lines AGS and MKN45 and mouse embryo fibroblast cell NIH-3 T3 (Institute of Cellular Research, Chinese Academy of Science, Shanghai, China) were maintained in RPMI-1640 medium supplemented with 10% fetal bovine serum (FBS). All the cells were incubated in a stable environment with 5% CO2 at 37°C in a humidified incubator (Thermo, Forma Scientific, Inc., USA).

### Transient transfection and luciferase assays

For transfection, NIH-3 T3, AGS and MKN45 cells were seeded onto 24-well plates (100,000 cells per well), and each well was cotransfected with 2.25 μg of the vector DNA with either SNP309T or SNP309G allele and internal control 10 ng pRL-SV40, in which a cDNA could encode the Renilla luciferase, and the pGL3-basic vector without any insert was applied as the negative control. After 24 h transfection, cells were lysed and luciferase activity was assayed by using the Dual-Luciferase Reporter Assay System (Promega) and normalized according to the Renilla luciferase activity. Each plasmid construct were done for independent triplicate.

### Purification and utilization of LPS

LPS was extracted from *H. pylori* strains (26695) using the phenol-water extraction procedure previously described [27], and was quantitated using the purpald assay [28].

AGS cells were cotransfected with either *MDM2* SNP309T or SNP309G luciferase reporters with Renilla luciferase reporter (as internal control) for 24 h, followed by incubation with two concentrations (0.1 and 1 μg/ml) of *H. pylori* LPS for 24 h. Cells were lysed and luciferase activity performed by using the Dual-Luciferase Reporter Assay System (Promega) and normalized according to the Renilla luciferase activity. Each plasmid construct were done for independent triplicate.

### Statistical analysis

Hardy-Weinberg equilibrium of alleles was evaluated by chi-square test. Comparison of age between cases and controls was evaluated by the Mann–Whitney U test, and the difference in the distribution of genotypes between cases and controls was determined by chi-square test. The association between the *MDM2* SNP309 and the risk for gastric carcinoma was estimated by odds ratios (OR) with the 95% confidence intervals (CI). Logistic regression was used to control for selected potential confounders (sex, age, and *H. pylori* infection) and to assess crude and adjusted OR and 95% CI. The difference in the levels of luciferase reporter gene expression between different constructs was examined by student’s test. Kaplan-Meier was used to construct the cumulative overall survival curves and the difference was tested by the log-rank test, and Cox’s proportional hazards model was used to perform the multivariate analysis to adjust for age, sex, TNM stage and *H. pylori*. All data analyses were done using SPSS software (version 11.0, Chicago, IL, USA). A *P* value of < 0.05 was considered statistically significant. Our sample size of 574 gastric carcinoma patients and 574 controls provided a 93% power to test an OR of 1.5 at the first error of 0.05 in a 2-sided test.

### Meta-analysis

We identified related studies by searching the PubMed, Embase, Web of Science, China National Knowledge Infrastructure and Wanfang databases up to August 2012 with the search phrases of “murine double minute or MDM2” and “polymorphism(s) or SNP309” and “gastric or stomach” and “cancer or carcinoma or tumor or neoplasm”. Additional studies were checked by screening reference lists of studies and reviews. The following criteria were used for literature selection: (a) the case–control study of the association between *MDM2* SNP309 polymorphism and gastric carcinoma susceptibility; (b) genotypic frequencies in both cases and controls available. Only the study of most recent and complete was used in this meta-analysis. Hence, we included 5 studies in our meta-analysis, containing 1935 cases and 2953 controls. The following information was collected from each publication: the first author’s name, year of publication, country, number of cases and controls, and *MDM2* genotype information.

The odds ratios (ORs) with 95% confidence intervals (CIs) were pooled to assess the association between *MDM2* SNP309 and gastric carcinoma risk. The fixed effect model (Mantel-Haenszel method) and the random effect model (the Dersimonian and Laird method) were used to pool data from different studies [29,30]. If the heterogeneity between studies is absent, the fixed effect model was used; otherwise, the random effect model was applied. The Z test was used to determine the statistical significance of the OR. The chi-square-based Q test and I^2^ were used to assess the statistical heterogeneity between studies, heterogeneity was considered statistically significant when *P* ≤ 0.05 [30], and Ι^2^ was used to qualify variation in OR attributable to heterogeneity. The publication bias was estimated by the Egger’s test [31]. *P* values < 0.05 are considered statistically significant. All analysis was done by using the Review Manage (v.4.2) and Statistical Analysis System software (v.9.1.3, SAS Institute, Cary, NC).

## Results

### Patient characteristics

The cases and controls were well matched on sex and age. The median age was 58.56 years (range, 21–90 years) for cases and 58.29 years (range, 22–88 years) for controls. There was no significance between cases and controls in sex distribution (Table 1). *H. pylori* infection was detected 408 (71.1%) of cases and 390 (67.9%) controls (Table 1).

**Table 1:** Demographic, clinical and pathological characteristics of study subjects

**Characteristics**	**Cases (n = 574)**	**Controls (n = 574)**	***P *value value**
Age (Mean ± SD, years)	58.56 ± 12.09	58.29 ± 11.88	0.71
Sex (Male/female)	399/175	399/175	1.00
Seropositive rate of *H. pylori *infection	71.1%	67.9%	0.25
Location of gastric carcinoma			
Cardia	181	NA	
Noncadia	393	NA	
Vascular invasion			
Absent	363	NA	
Present	211	NA	
Lymph node metastasis			
Absent	173	NA	
Present	401	NA	
Liver metastasis		NA	
Absent	512	NA	
Present	62	NA	
Peritoneal dissemination			
Absent	503	NA	
Present	71	NA	
TNM stage			
IA	96	NA	
IB	79	NA	
II	107	NA	
III	173	NA	
IV	119	NA	
Follow-up (months)		NA	
Median (Range)	36 (4–48)	NA	

Abbreviations: *SD*, standard deviation; *NA*, not applicable.

### Association between MDM2 promoter SNP309 polymorphism and gastric carcinoma

The genotype frequencies of the controls (n = 574) were consistent with the Hardy-Weinberg equilibrium distribution (*P* = 0.98).

The genotype distributions in *MDM2* polymorphism of cases and controls are shown in Table 2. The *MDM2* SNP309 genotype frequencies were 30.1% (T/T), 45. 3% (T/G) and 24.6% (G/G) in cases, and 34.7% (T/T), 51.6% (T/G) and 13.7% (G/G) in control subjects (*P* < 0.01). The SNP309G/G genotype, but not SNP309T/G genotype, was associated with an increased risk of gastric carcinoma; the adjusted OR (95%CI) was 2.06 (1.46-2.90) and 1.01 (0.78-1.31) for SNP309G/G homozygotes and SNP309T/G heterozygotes, respectively, compared with the SNP309T/T homozygotes. In the recessive model, the SNP309G/G homozygote was associated with a 2.05-fold increased risk of gastric carcinoma, compared with the T carriers (adjusted OR, 2.05; 95% CI, 1.51-2.78; *P* < 0.01). Table 3 shows a significantly increased risk of gastric carcinoma in patients with SNP309G/G genotype and *H. pylori* infections (adjusted OR, 2.44, which was greater than 1.14 × 1.94 = 2.21), compared with T carriers without *H. pylori* infection.

**Table 2:** **The genotype distributions of *MDM2 *SNP309 in the cases with gastric carcinoma and healthy controls**

	**Controls (n = 574)**	**Patients (n = 574)**	**Crude OR (95% CI)**	***P *value**	**Adjusted OR (95% CI)**^**†**^	***P *value**
Genotype						
T/T	34.7%	30.1%	1.00			
T/G	51.6%	45.3%	1.01 (0.78-1.32)	0.94	1.01 (0.78-1.31)	0.95
G/G	13.7%	24.6%	2.05 (1.46-2.89)	< 0.01	2.06 (1.46-2.90)	< 0.01
Recessive model						
T carriers	86.3%	75.4%	1.00			
G/G	13.7%	24.6%	2.04 (1.51-2.77)	< 0.01	2.05 (1.51-2.78)	< 0.01
Dominant model						
T/T	31.2%	30.1%	1.00			
G carriers	68.8%	69.9%	1.23 (0.96-1.58)	0.10	1.23 (0.96-1.58)	0.10

^†^Adjusted for age, sex, and *H. pylori *infection.

Abbreviations: *OR*, odds ratio; *CI*, confidence interval.

**Table 3:** **Association between *MDM2 *SNP309 and gastric carcinoma in relation to *H. pylori *infection**

**MDM2**	***H. pylori *infection**	**Odds ratio**^**† **^**(95% Confidence interval)**	***P *value**
T carriers	Negative	1.00	
T carriers	Positive	1.14 (0.86-1.51)	0.37
GG	Negative	1.94 (1.11-3.38)	0.02
GG	Positive	2.44 (1.62-3.66)	< 0.01

^†^Adjusted for age and sex.

### Association of MDM2 SNP309 and gastric carcinoma in relation to the location, metastatic status and TNM stage of gastric carcinoma

The risks of subtypes of gastric carcinoma for SNP309G/G were significantly increased when compared with T carriers, especially non-cardiac carcinoma (adjusted OR, 2.18; 95% CI, 1.57-3.04), lymph node metastasis present carcinoma (adjusted OR, 2.25; 95% CI, 1.63-3.12), liver metastasis present carcinoma (adjusted OR, 3.34; 95% CI, 1.84-6.08), peritoneal dissemination present carcinoma (adjusted OR, 2.13; 95% CI, 1.18-3.84), and advanced stages carcinoma (adjusted OR, 2.30; 95% CI, 1.52-3.49 for stage III, and 2.29; 95% CI, 1.42-3.69 for stage IV; Table 4).

**Table 4:** **Association between *MDM2 *SNP309 and gastric *carcinoma *in relation to location, metastasis and TNM stage**

**Variable**	***MDM2 *SNP309 genotype (Control/Case)**		**Adjusted OR (95% CI) **^**†**^		***P *value**
	**TT + TG**	**GG**	**TT + TG**	**GG**	
Location					
Cardia	495/141	79/40	1.00	1.84 (1.20-2.83)	< 0.01
Non-cardia	495/292	79/101	1.00	2.18 (1.57-3.04)	< 0.01
Vascular invasion					
Absent	495/265	79/98	1.00	2.33 (1.67-3.26)	< 0.01
Present	495/168	79/43	1.00	1.60 (1.06-2.42)	0.03
Lymph node metastasis			1.00		
Absent	495/138	79/35	1.00	1.59 (1.02-2.47)	0.04
Present	495/295	79/106	1.00	2.25 (1.63-3.12)	< 0.01
Liver metastasis			1.00		
Absent	495/391	79/121	1.00	1.94 (1.42-2.65)	< 0.01
Present	495/42	79/20	1.00	3.34 (1.84-6.08)	< 0.01
Peritoneal dissemination					
Absent	495/380	79/123	1.00	2.04 (1.49-2.79)	< 0.01
Present	495/53	79/18	1.00	2.13 (1.18-3.84)	0.01
TNM stage					
IA	495/75	79/21	1.00	1.75 (1.01-3.00)	0.04
IB	495/60	79/19	1.00	2.01 (1.13-3.57)	0.01
II	495/83	79/24	1.00	1.78 (1.06-2.98)	0.03
III	495/127	79/46	1.00	2.30 (1.52-3.49)	< 0.01
IV	495/88	79/31	1.00	2.29 (1.42-3.69)	< 0.01

^†^Adjusted for age, sex, and *H. pylori *infection.

Abbreviations: *OR*, odds ratio; *CI*, confidence interval.

### Kaplan-Meier survival cures

**Figure 1:**
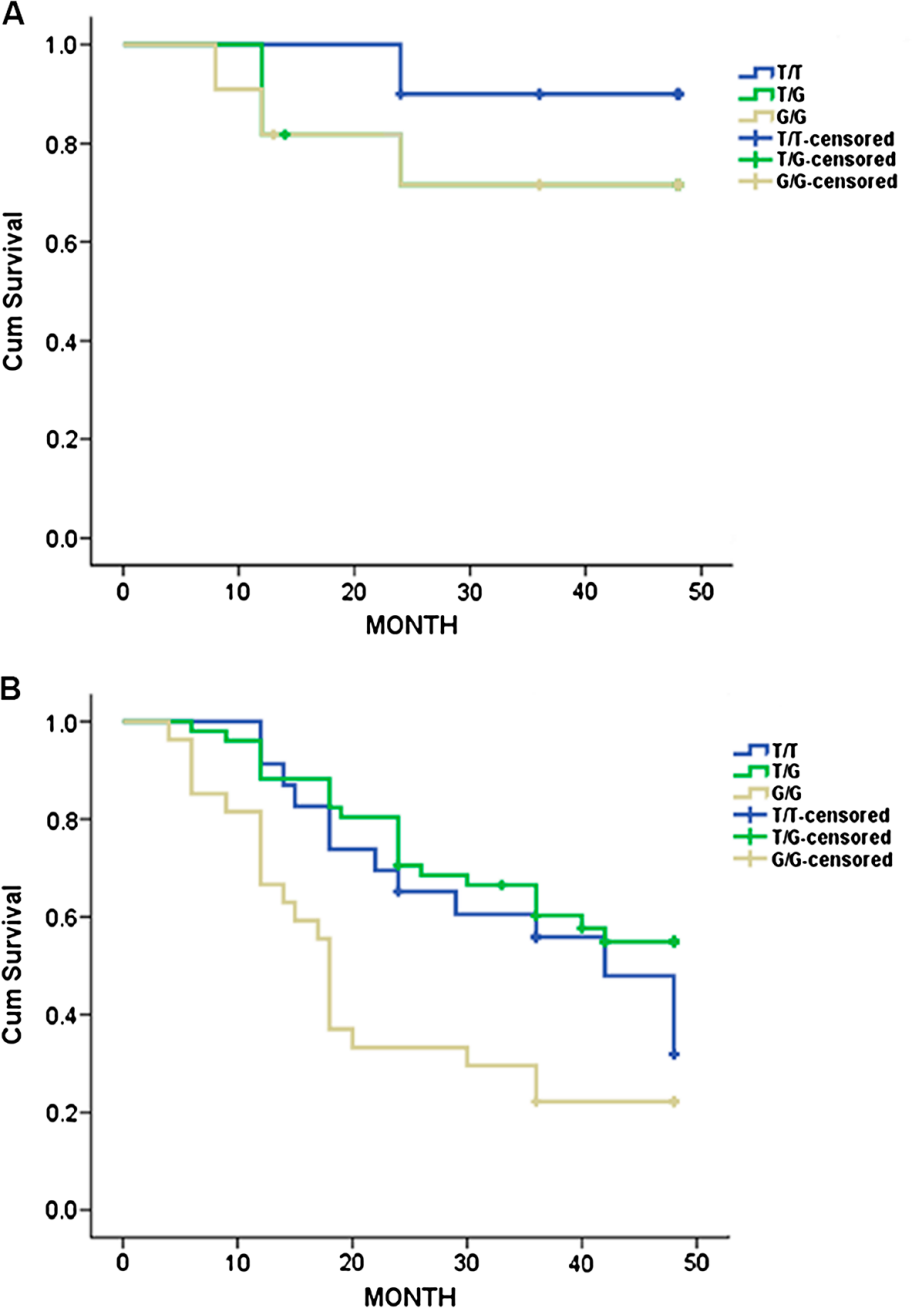
**Association between MDM2 SNP309 and survival of patients with gastric carcinoma according to TNM classification. **(**A**) Kaplan-Meier survival curves of 32 patients with TNM I stage according to MDM2 SNP309 genotypes (*P* = 0.50). (**B**) Kaplan-Meier survival curves of 101 patients with TNM II-IV stages according to MDM2 SNP309 genotypes (*P* < 0.01).

Overall, 133 patients with gastric carcinoma were followed up for a median (range) of 36 (4–48) months. Of the 101 patients with TNM stages II (T1N2-N3M0, T2N1-N2M0, T3N0-N1M0, or T4aN0M0) to IV (TanyNanyM1), SNP309 (G/G) homozygotes had a significant association of poor overall survival (*P* < 0.01, Figure 1B). The difference in survival among SNP309 genotypes in 32 stage I (T1N0-N1M0 or T2N0M0) patients was not significant (*P* = 0.50, Figure 1A). In addition, Cox proportional hazards analysis showed that SNP309 (G/G) was an independent factor for poor prognosis of gastric carcinoma with TNM II -IV stages (hazard ratio, 2.13; 95% CI, 1.04 to 4.36; *P* = 0.04) to adjust for sex, age, TNM stage and *H. pylori* infection.

### Effects of MDM2 SNP309 (T > G) on transcriptional activity of MDM2 gene

**Figure 2:**
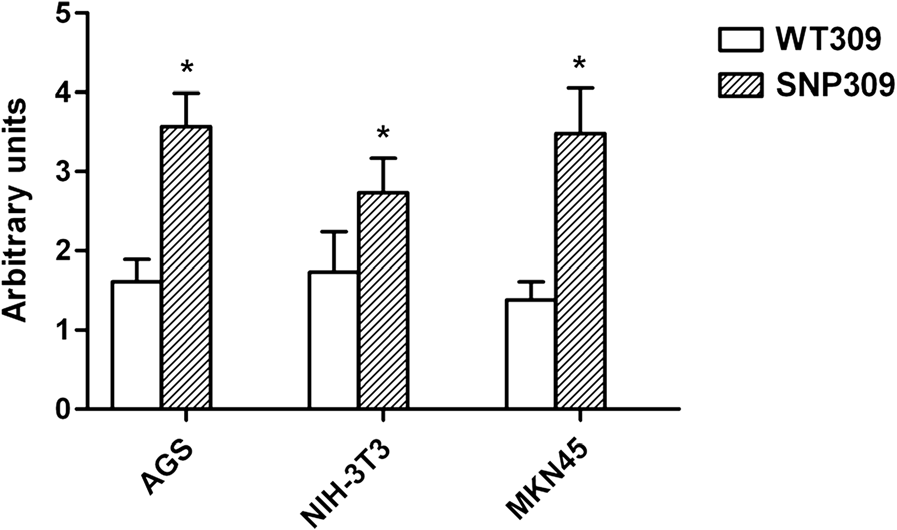
**Effect of MDM2 SNP309 in the transcriptional activity of MDM2 promoter. **Data are expressed as mean and standard deviation of three independent experiments. WT309, cells transfected with allele 309 T; SNP309, cells transfected with allele 309 G; * *P* < 0.01, compared with WT309.

**Figure 3:**
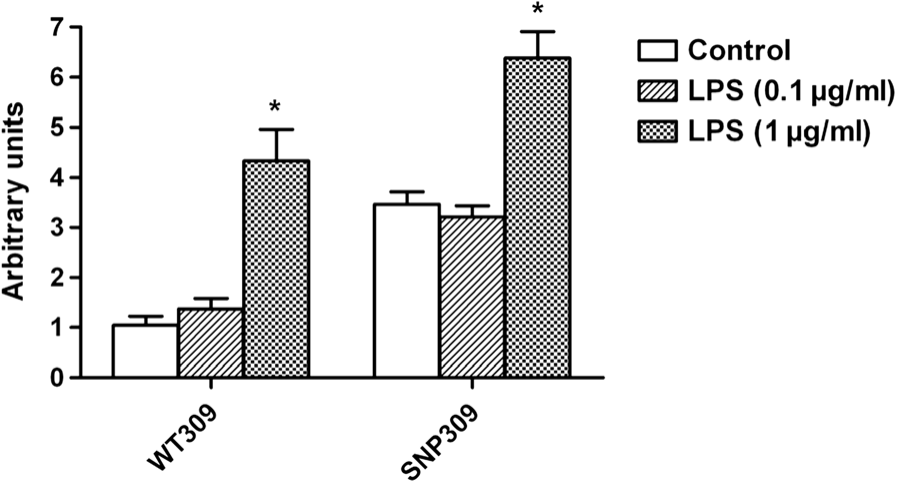
**Combined effect of transfection with MDM2 SNP309 (T > G) polymorphism and H. pylori lipopolysaccharide (LPS) on transcriptional activity of MDM2 in AGS cell. **WT309, cells transfected with allele 309 T; SNP309, cells transfected with allele 309 G; * *P* < 0.01, compared with WT309 or SNP309, where appropriate.

As shown in Figure 2, the vectors with SNP309G allele produced a 100% to 300% increase in the relative luciferase activities, compared with that of SNP309T allele in all three types of cell lines (all *P* < 0.01). Furthermore, *H. pylori* LPS 1 μg/ml significantly increased the relative luciferase activity, compared with untreated controls in AGS cell, no matter whether SNP309T or SNP309G allele was transfected (*P* < 0.01, Figure 3).

### Meta-analysis of association between MDM2 promoter SNP309 polymorphism and gastric carcinoma

We identified 4 published studies of the *MDM2* SNP309 and gastric carcinoma susceptibility. With the combined data from those previous studies and from our current study, this meta-analysis included 1935 cases and 2953 controls. The characteristics of these studies are summarized in Additional file 1: Table S1. The studies are all Asian populations. As shown in Additional file 2: Table S2 and Additional file 3: Table S3, compared with the SNP309T/T genotype, the SNP309G/G genotype was associated with a significant increased risk of gastric carcinoma (OR, 1.57; 95% CI, 1.08-2.29; *P* = 0.02), whereas the SNP309T/G genotype was not associated significant with the risk of gastric carcinoma (OR, 1.08; 95% CI, 0.83- 1.41; *P* = 0.57). In the recessive model, the SNP309G/G homozygote was associated with a 1.51 -fold increased risk of gastric carcinoma, compared with the T carriers (95% CI, 1.21-1.89; *P* < 0.01). However, in the dominant model, the G carriers was associated with a 1.28-fold increased risk of gastric carcinoma, compared with the SNP309T/T genotype (95% CI, 0.88-1.86; *P* = 0.19). All stratified analyses are based on the recessive model. When stratified analysis by location of gastric carcinoma, the SNP309G/G homozygote was associated with a increased risk of gastric carcinoma, both in cardiac (OR, 1.65; 95% CI, 1.12-2.43; *P* = 0.01) and non- cardiac gastric carcinoma (OR, 1.78; 95% CI, 1.42-2.23; *P* < 0.01) compared with the T carriers. Stratified by the histologic subtype, the SNP309G/G homozygote was associated with a 1.37-fold significant increased risk of intestinal carcinoma (95% CI, 1.04-1.81; *P* = 0.03), but not the diffuse carcinoma (OR, 1.05; 95% CI, 0.76-1.43; *P* = 0.78).

The publication bias of included studies was assessed by the Egger’s test, and the results did not show evidence of publication bias (t, 1.42; *P* = 0.25 for T/G vs T/T, t, 2.62; *P* = 0.08 for G/G vs T carriers, and t, 2.08; *P* = 0.13 for G carriers vs TT) except for G/G vs T/T (t, 3.40; *P* = 0.04).

## Discussion

It has been reported that the *MDM2* SNP309G variant is bound more efficiently by Sp1 than 309 T allele, which increases MDM2 protein expression levels and weakens the p53 function [7]. So far, no consistent conclusion has been reached on the association between *MDM2* polymorphism and gastric carcinoma risk [19-23], and there has been only one study reporting that *MDM2* polymorphism is associated with both an increased susceptibility and poor prognosis of gastric carcinoma [19]. In our present large case–control analysis, we found that the SNP309G/G genotype was significant associated with gastric carcinoma risk and poor clinical prognosis. In addition, our meta-analysis also indicated that the *MDM2* SNP309G/G genotype was associated significantly with an increased risk of gastric carcinoma, and provided further evidence indicating an association between this functional polymorphism and gastric carcinoma susceptibility. We also showed that the T to G substitution of this polymorphism significantly enhanced the transcription activity of the *MDM2* gene in vitro.

It has been reported that *MDM2* SNP309 is associated with poor prognosis of different kinds of carcinomas [32-34], and that SNPG/G is associated with shortened survival of patients with advanced gastric carcinoma [19]. In agreement with previous studies, we also found *MDM2* SNP309G/G independently predicted poor prognosis of gastric carcinoma.

Intriguingly, we found that individuals with both *MDM2* SNP309G/G and *H. pylori* infection conferred a synergistic effect for developing gastric carcinoma with an OR of 2.44, suggesting a joint effect between *MDM2* polymorphism and *H. pylori* infection. *H. pylori* itself has been identified as the cause of gastric carcinoma and was classified as a Class I human carcinogen by the WHO [12,35-37]. It has reported that *H. pylori* infection increases the expression of MDM2 protein in the gastric mucosa [38,39], and the overexpression inhibits p53 function of tumor suppression [5]. It has been shown that *H. pylori* is able to phosphorylate and active MDM2 and subsequent degradation of p53 by activating AKT1 in gastric epithelial cells [40]. In addition, the cag PAI also contributes to p53 inactivation because individuals infected with *H pylori cagA* strains have a higher likelihood of harboring p53 mutations [41]. Therefore, individuals with both *MDM2* SNP309G/G genotypes and *H. pylori* infection are expected to have a higher risk in development of gastric carcinoma.

The association of *H. pylori* vacuolating cytotoxin A (VacA) or *cytotoxin associated gene A protein* (CagA) with gastric carcinoma has been extensively reported [42-44]. Recent studies have reported that LPS, another virulence factor of *H. pylori*, is closely related to gastric carcinoma [16,17]. Like E. coli LPS, *H. pylori* LPS is also composed of the specific polysaccharide, core polysaccharide and lipid A composition, and it has a certain toxicity and immune regulation, immune stimulation. Compared with E. coli LPS, *H. pylori* LPS has a lower endotoxin activity, and the ability of stimulating macrophages to produce pro-inflammatory factors and the role of nitric oxide are all weaker; however, it is the weak activity to endotoxin that sustains chronic gastric inflammation [45,46]. In the present study, we found that *H. pylori* LPS elevated the transcriptional activity of the gene *MDM2*, both in *MDM2* WT309T and SNP309G. It has been reported that *H. pylori* LPS promotes colonization of *H. pylori* in the mucus layer adjacent to the gastric epithelial surface by interacting specifically with trefoil factor 1 in the stomach [15], and enhances proliferation and progression of gastric carcinoma by attenuating the antitumor activity and IFN- γ -mediated cellular immunity [16,17]. Our data suggested that the synergistic effect by *MDM2* SNP309 (T > G) polymorphism and *H. pylori* infection may attribute to *H. pylori* LPS although further investigation is required.

## Conclusion

In conclusion, the *MDM2* SNP309 polymorphism is associated with gastric carcinoma risk and poor prognosis in Chinese patients. In addition, there is a joint effect of *MDM2* SNP309G/G allele and *H. pylori* infection for the development of gastric carcinoma, which may attribute to *H. pylori* LPS.

## Competing interests

The authors declare that they have no competing interests.

## Authors’ contribution

GXZ XYW and RHS conceived and designed the experiments. XLP YQL JF and BH performed the experiments. XLP YQL and JF analyzed the data. GXZ contributed reagents/materials/analysis tools. XLP and YQL wrote the paper. All authors read and approved the final manuscript.

## Pre-publication history

The pre-publication history for this paper can be accessed here:

http://www.biomedcentral.com/1471-2407/13/126/prepub

## Supplementary Material

Additional file 1: Table S1 Characteristics of studies included in the current meta-analysis. Click here for file

Additional file 2: Table S2 Result of meta-analysis of the association between *MDM2 *SNP309 polymorphism and gastric carcinoma risk. Click here for file

Additional file 3: Table S3 Stratified of meta-analysis of the association between *MDM2 *SNP309 polymorphism and gastric carcinoma risk. Click here for file
