# Supplementary material for: A functional polymorphism T309G in MDM2 gene promoter, intensified by Helicobacter pylori lipopolysaccharide, is associated with both an increased susceptibility and poor prognosis of gastric carcinoma in Chinese patients
Source: BMC Cancer. 2013 Mar 18;13:126. doi: 10.1186/1471-2407-13-126 (PMC3621260; doi:10.1186/1471-2407-13-126)
Supplement: Additional file 1: Table S1 — Characteristics of studies included in the current meta-analysis. [file 1471-2407-13-126-S1.doc]

**Table S1 Characteristics of studies included in the current meta-analysis**

|  |  | Cases | | | Controls | | |
| --- | --- | --- | --- | --- | --- | --- | --- |
| Study | Country | T/T | T/G | G/G | T/T | T/G | G/G |
| Ohmiya 2006 [19] | Japan | 98 | 188 | 124 | 99 | 241 | 98 |
| Yang 2007 [20] | China | 107 | 250 | 143 | 298 | 498 | 204 |
| Cao 2007 [21] | China | 21 | 91 | 100 | 117 | 299 | 226 |
| Cho 2008 [22] | Korea | 64 | 110 | 65 | 61 | 152 | 86 |
| Current study | China | 173 | 260 | 141 | 199 | 296 | 79 |
