# Supplementary material for: A functional polymorphism T309G in MDM2 gene promoter, intensified by Helicobacter pylori lipopolysaccharide, is associated with both an increased susceptibility and poor prognosis of gastric carcinoma in Chinese patients
Source: BMC Cancer. 2013 Mar 18;13:126. doi: 10.1186/1471-2407-13-126 (PMC3621260; doi:10.1186/1471-2407-13-126)
Supplement: Additional file 2: Table S2 — Result of meta-analysis of the association between MDM2 SNP309 polymorphism and gastric carcinoma risk. [file 1471-2407-13-126-S2.doc]

**Table S2 Result of meta-analysis of the association between *MDM2* SNP309 polymorphism and gastric carcinoma risk**

| Comparison Model | *P* for Heterogeneity | OR | 95% CI | *P* |
| --- | --- | --- | --- | --- |
| T/G vs T/T | 0.02 | 1.08 | 0.83-1.41 | 0.57 |
| G/G vs T/T | < 0.01 | 1.57 | 1.08-2.29 | 0.02 |
| G/G vs T carriers | 0.30 | 1.51 | 1.21-1.89 | < 0.01 |
| G carriers vs T/T | < 0.01 | 1.28 | 0.88-1.86 | 0.19 |

Abbreviations: OR, odds ratio; CI, confidence interval.
