# Supplementary material for: A functional polymorphism T309G in MDM2 gene promoter, intensified by Helicobacter pylori lipopolysaccharide, is associated with both an increased susceptibility and poor prognosis of gastric carcinoma in Chinese patients
Source: BMC Cancer. 2013 Mar 18;13:126. doi: 10.1186/1471-2407-13-126 (PMC3621260; doi:10.1186/1471-2407-13-126)
Supplement: Additional file 3: Table S3 — Stratified of meta-analysis of the association between MDM2 SNP309 polymorphism and gastric carcinoma risk. [file 1471-2407-13-126-S3.doc]

**Table S3 Stratified of meta-analysis of the association between *MDM2*** SNP309 polymorphism and gastric carcinoma risk

|  | G/G vs T Carriers | | |
| --- | --- | --- | --- |
| Variable | OR | 95% CI | *P* |
| Location site |  |  |  |
| Cardia | 1.65 | 1.12-2.43 | 0.01 |
| Non-cardia | 1.78 | 1.42-2.23 | < 0.01 |
| Histotype |  |  |  |
| Intestinal | 1.37 | 1.04-1.81 | 0.03 |
| Diffuse | 1.05 | 0.76-1.43 | 0.78 |

Abbreviations: OR, odds ratio; CI, confidence interval.
